# De Leefstijlmonitor: Cijfers voor gezondheidsbeleid

**DOI:** 10.1007/s12508-022-00349-8

**Published:** 2022-05-13

**Authors:** Ellen de Hollander, Christianne Hupkens, Saskia van Dorsselaar, Wanda Wendel-Vos, Ellen Kemler, Hanneke de Graaf, Annette Stafleu, Karen Hosper, Nannah Tak, Caroline van Rossum, Koenraad Vermey, Marieke Hiemstra

**Affiliations:** 1 Rijksinstituut voor Volksgezondheid en Milieu, Centrum Voeding Preventie en Zorg, Bilthoven, Nederland; 2 Trimbos-instituut, Utrecht, Nederland; 3 VeiligheidNL, Amsterdam, Nederland; 4 Rutgers, Utrecht, Nederland; 5 Soa Aids Nederland, Amsterdam, Nederland; 6 Voedingscentrum, Den Haag, Nederland; 7 Pharos, Utrecht, Nederland; 8 GGD GHOR Nederland, Utrecht, Nederland; 9 Centraal Bureau voor de Statistiek, Heerlen, Nederland

**Keywords:** leefstijlmonitor, roken, alcohol, voeding, gezondheidsbeleid, Lifestyle monitor, Smoking, Alcohol, Nutrition, Health policy

## Abstract

In 2013 is in opdracht van het Ministerie van Volksgezondheid, Welzijn en Sport de Leefstijlmonitor (LSM) gestart om de gegevensverzameling op het gebied van leefstijl in Nederland efficiënter en meer samenhangend te organiseren om te komen tot eenduidige cijfers voor gezondheidsbeleid. Dit artikel beschrijft het ontstaan, de verschillende onderdelen en de thema’s van de LSM, en de taken en rollen van de samenwerkende partijen. Daarnaast beschrijft het hoe de gegevens verzameld worden en waar de gegevensverzamelingen op te vragen zijn voor onderzoek. Tot slot bevat het enkele voorbeelden van cijfers en trends over de periode 2014–2020 ter ondersteuning van gezondheidsbeleid**.**

## Abstract (en)

The Lifestyle Monitor (LSM) was launched in 2013 on behalf of the Ministry of Health, Welfare and Sports to reorganize the multiple data collections in the field of lifestyle and health in the Netherlands. The reorganization should enhance the efficiency and coherence of the lifestyle and health data collections and should provide unambiguous figures for policymakers. This article describes the background and content (including the lifestyle-themes) of the LSM and the tasks and roles of the collaborating parties involved. The measurement methods used and requirements for data requests are described as well. Finally, some examples of figures and trends over the period 2014–2020 to underpin the health policy are described.

## Hoe is de Leefstijlmonitor tot stand gekomen?

Voor de onderbouwing van nationaal gezondheidsbeleid is een monitoringssysteem van belang waarin cijfers over leefstijl en gezondheid worden gemonitord over tijd. Deze cijfers worden vaak verzameld via vragenlijstonderzoek in een steekproef van de bevolking. In Nederland kennen we een geschiedenis van diverse vragenlijstonderzoeken die zich vaak richtten op één onderwerp en worden uitgevoerd door thema-instituten, zoals het Continu Onderzoek Rookgewoonten (COR) van Stivoro/het Trimbos-instituut. Daarnaast waren er onderzoeken die zich richtten op meerdere onderwerpen, waaronder leefstijlgedragingen, zoals de Gezondheidsenquête (GE) van het Centraal Bureau voor de Statistiek (CBS) [1].

In 2011 startte het ministerie van Volksgezondheid, Welzijn en Sport (VWS) met de herijking van het leefstijlbeleid. Een van de vragen die daaruit volgde was of de verschillende gegevensverzamelingen over leefstijl efficiënter en in samenhang met elkaar ingericht konden worden [2]. Het ministerie heeft het Landelijk Overleg Thema instituten (LOT-i) destijds om advies gevraagd over welke minimale basisinformatie beschikbaar zou zijn voor de informatiebehoefte van de overheid en welke bestaande bronnen daarvoor zouden kunnen dienen. Het LOT‑i adviseerde om onder regie van het Rijksinstituut van Volksgezondheid en Milieu (RIVM) een plan van aanpak uit te werken, in samenwerking met de thema-instituten, CBS, Gemeentelijke Gezondheidsdiensten (GGD’en) en het Sociaal Cultureel Planbureau (SCP) [3]. In 2012 publiceerde het RIVM een adviesrapport waarin werd voorgesteld de gegevensverzamelingen beter af te stemmen en meer te bundelen, dubbelingen te verwijderen en de frequentie van sommige onderzoeken te verlagen door middel van een samenwerking tussen de betrokken partijen [1], om zo te zorgen voor kwaliteitsverbetering en efficiencywinst.

In 2013 en 2014 werd dit voorstel uitgewerkt tot een vernieuwde opzet van de verschillende monitoringsactiviteiten onder de noemer ‘de Leefstijlmonitor’, ondersteund door het ministerie van VWS. Het doel van de Leefstijlmonitor (LSM) is het efficiënter en meer samenhangend organiseren van de landelijke gegevensverzameling op het gebied van leefstijl in Nederland [4]. Binnen de LSM wordt onderscheid gemaakt tussen de kern van de Leefstijlmonitor (LSM-K) en de aanvullende modules van de Leefstijlmonitor (LSM-A). De LSM‑K dient als basis voor leefstijlbeleid waarbinnen de belangrijkste gegevens (twee)jaarlijks worden verzameld om trends in de tijd te volgen. De LSM‑A wordt minder frequent uitgevoerd dan de LSM‑K en biedt informatie die aanvullend is op de LSM‑K, en kan gebruikt worden om verdieping te zoeken binnen een thema. Dit artikel beschrijft wat de LSM inhoudt en geeft enkele voorbeelden van cijfers en trends over de periode 2014–2020 ter illustratie van wat de LSM aan cijfers oplevert.

## Wat houdt de LSM in?

### Wie is er betrokken bij de LSM?

De LSM is een product van de samenwerking tussen partijen die zich richten op leefstijl. De volgende partijen vormen samen het Consortium LSM: Trimbos-instituut, Rutgers, Soa Aids Nederland, Pharos, VeiligheidNL, Voedingscentrum, CBS en GGD GHOR Nederland. Het RIVM fungeert binnen de LSM als coördinator van 1) de LSM, 2) het netwerk kernindicatoren sport en bewegen en 3) de VoedselConsumptiePeiling (VCP). De genoemde partijen zijn samen verantwoordelijk voor de inhoud van de LSM en publiceren cijfers voor gezondheidsbeleid op basis van de gegevensverzamelingen van de LSM. De taken van de partijen en hun rollen binnen de LSM staan beschreven in tab. 1.

**Table 1:** 

partij	taak	rol in de Leefstijlmonitor
Trimbos-instituut	onafhankelijk kennisinstituut voor alcohol, tabak, drugs en mentale gezondheid	1. partij van het consortium vanwege themagerelateerde activiteiten op het gebied van middelengebruik 2. uitvoerder van HBSC (binnen HBSC-NL) en PEIL
Rutgers	expertisecentrum gericht op seksuele en reproductieve gezondheid en rechten	1. partij van het consortium vanwege themagerelateerde activiteiten op het gebied van seksuele gezondheid 2. uitvoerder van Seks onder je 25e
Soa Aids Nederland	expertisecentrum gericht op het succesvol voorkomen, opsporen en behandelen van hiv en andere soa’s	1. partij van het consortium vanwege themagerelateerde activiteiten op het gebied van seksuele gezondheid 2. uitvoerder van Seks onder je 25e en Seksuele Gezondheid in Nederland
Pharos	expertisecentrum gericht op het terugdringen van sociaaleconomische gezondheidsverschillen	partij van het consortium vanwege aandacht voor inclusief onderzoek en monitoring van sociaaleconomische verschillen in leefstijl
VeiligheidNL	kenniscentrum voor letselpreventie	partij van het consortium vanwege themagerelateerde activiteiten op het gebied van sportblessures en ongevallen
Voedingscentrum	kenniscentrum gericht op een gezonde, veilige en meer duurzame voedselkeuze	partij van het consortium vanwege themagerelateerde activiteiten op het gebied van voeding
CBS	statistisch bureau van Nederland dat gegevens verzamelt over Nederland en de Nederlandse samenleving	1. partij van het consortium vanwege themagerelateerde activiteiten op het gebied van leefstijl en gezondheid 2. coördinator en eigenaar van de Gezondheidsenquête, waarbinnen een groot deel van de gegevensverzameling van LSM‑K valt 3. uitvoerder van een deel van de gegevensverzamelingen van LSM‑A
GGD GHOR Nederland	vereniging voor publieke gezondheid en veiligheid in Nederland; overkoepelende brancheorganisatie van de 25 GGD’en en GHOR	1. partij van het consortium vanwege themagerelateerde activiteiten in het kader van de Gezondheidsmonitor 2. harmonisatie tussen de Gezondheidsmonitor en de LSM‑K bevorderen waarbij de belangen van de GGD’en worden behartigd
RIVM	kennis- en onderzoeksinstituut gericht op de bevordering van de volksgezondheid en een gezond en veilig leefmilieu	1. coördinator van de Leefstijlmonitor en voorzitter van het consortium 2. partij van het consortium vanwege themagerelateerde activiteiten op het gebied van leefstijl en gezondheid 3. coördinator en uitvoerder van VCP 4. coördinator van het Netwerk Kernindicatoren Sport en Bewegen

*HBSC* Health Behaviour in School-aged Children study, *PEIL* Peilstationsonderzoek Scholieren, *VCP* VoedselConsumptiePeiling, *LSM‑K* kern van de Leefstijlmonitor, *LSM‑A* aanvullende modules van de Leefstijlmonitor, *GGD’en* Gemeentelijke Gezondheidsdiensten, *GHOR* Geneeskundige Hulpverleningsorganisaties in de Regio

### Wat wordt er gemeten met de LSM?

De thema’s die onder de LSM vallen betreffen de thema’s waarop de thema-instituten zich richten en waarvan een relatie met gezondheid is vastgesteld [1, 4], namelijk:

- roken;
- alcoholgebruik;
- drugsgebruik (inclusief sportprestatieverhogende middelen);
- seksuele gezondheid;
- bewegen, sport en zitten;
- voeding;
- gezond gewicht;
- ongevallen (inclusief sportblessures).

### Welke gegevensverzamelingen vallen onder de LSM?

De LSM maakt gebruik van verschillende gegevensverzamelingen sinds 2014. Binnen de LSM zijn op basis van methodologische overwegingen afspraken gemaakt over welke gegevensverzameling de voorkeur heeft voor het vergaren van cijfers voor gezondheidsbeleid binnen een bepaald thema (‘preferente gegevensverzameling’). Tabel 2 bevat een overzicht van kenmerken van deze gegevensverzamelingen en de bijbehorende thema’s. Details van de onderzoeksmethoden van de verschillende gegevensverzamelingen zijn te vinden via de webpagina ‘Onderzoeksbeschrijvingen’ op www.leefstijlmonitor.nl [5].

**Table 2:** 

Bron	Leeftijdsgroep	N (aantal deelnemers)	Responspercentage	Frequentie en startjaar	Leefstijlonderwerpen in vragenlijst	Betrokken consortium-partijen
Gezondheidsenquête (GE)	0 jaar en ouder (afhankelijk van onderwerp)	2014: 9.516 2015: 9.358 2016: 9.165 2017: 9.826 2018: 10.043 2019: 9.778 2020: 8.718	2014: 61,7% 2015: 61,2% 2016: 61,0% 2017: 57,7% 2018: 55,8% 2019: 54,3% 2020: 51,5%	sinds 1981 jaarlijks; Sinds 2014 als onderdeel van LSM 1 × in de 5/6 jaar internationale verplichting binnen het EHIS-onderzoek: 2014, 2019	lengte en gewicht, sport en bewegen (vanaf 2016 vanaf 4 jaar; 2014 en 2015 vanaf 12 jaar), voeding, ongevallen en sportblessures, roken, alcohol, drugsgebruik, seksuele gezondheid	alle
Peilstationsonderzoek Scholieren (PEIL)	12–16 jaar	2015: 7.244 voortgezet onderwijs (alle leerjaren vmbo, havo, vwo)^a^ 2019: 6,118 voortgezet onderwijs^a^	2015: 43,3% voortgezet onderwijs 2019: 40% voortgezet onderwijs	sinds 2003 1 × in de 4 jaar; sinds 2015 als onderdeel van LSM	roken, alcohol, drugs, seksueel gedrag	Trimbos-instituut, RIVM, Rutgers
Health Behaviour in Schoolaged Children (HBSC)	12–16 jaar	2017: 6.718 voortgezet onderwijs^a^	2017: 37% voortgezet onderwijs	sinds 2001 1 × in de 4 jaar; sinds 2017 als onderdeel van LSM	roken, alcohol, drugs, seksueel gedrag	Trimbos-instituut, RIVM, Rutgers
LSM‑A Middelen	15 jaar en ouder	2016: 10.706 2018: 10.348 2020: 9.789	2016: 57% 2018: 54% 2020: 49%	sinds 2016 1 × in de 2 jaar	roken (onder andere verslaving, verkooppunten, stoppogingen, meeroken, rookproducten); alcohol (onder andere verslaving, producten, attitude); drugs (onder andere producten, effecten van drugs); sportprestatieverhogende middelen	CBS, RIVM, Trimbos-instituut, Coördinator netwerk kernindicatoren sport en bewegen
LSM‑A Bewegen en Ongevallen	0 jaar en ouder (afhankelijk van onderwerp)	2015: 10.655 2017: 10.086 2019: 9.981	2015: 55% 2017: 59% 2019:47%	sinds 2015 1 × in de 2 jaar	sport en bewegen kinderen 0–3 jaar (vanaf 2019); sport en bewegen 4 jaar en ouder (inclusief seizoensgebonden sporten); onder andere tevredenheid aanbieder, zitgedrag; vanaf 4 jaar, ongevallen (onder andere type letsels, verzuim, context van het ongeval) sportblessures (onder andere type blessures en preventie blessures)	CBS, RIVM, VeiligheidNL, Coördinator netwerk kernindicatoren sport en bewegen
LSM‑A Seks onder je 25e	12–24 jaar	2017: 20.500	25,7%	sinds 2017 1 × in de 5 jaar; als onderdeel van LSM	verliefdheid en relaties, seksuele geaardheid, mening over seksueel gedrag, kennis over seks, ervaringen, algemene seksvragen, eerste keer seks, laatste sekspartner, opvattingen over seks, media, condooms, soa (voorkomen van), zwangerschap, kinderen en kinderwens, seksuele problemen, grensoverschrijding, ruilseks en betaalde seks	Rutgers, SOA Aids NL, GGD’en, RIVM
LSM‑A Seksuele gezondheid in Nederland	18 jaar en ouder	2017: ruim 17.000	21,5%	sinds 2017 1 × in de 5 jaar; als onderdeel van LSM	relaties, seksuele geaardheid, mening over seksueel gedrag, ervaringen, laatste sekspartner, opvattingen over seks, soa/hiv, (voorkomen van) zwangerschap, seksuele problemen, betaalde seks, online ervaringen, menopauze, kinderen en kinderwens, grensoverschrijding	Rutgers, RIVM
VoedselConsumptiePeiling (VCP) twee 24-uursvoedingsnavragen op niet-aaneengesloten, onafhankelijke dagen	1–79 jaar	2012–2016: 4.313	65%	sinds 2012 1 × in de 6 jaar; 2012–2016 als onderdeel van LSM	voedselconsumptie gemeten met behulp van twee 24-uursvoedingsnavragen op niet-aaneengesloten, onafhankelijke dagen (*dietary recall*); dit houdt in dat de inname van macro- en microvoedingsstoffen kunnen worden geschat	RIVM (coördinator VCP, en coördinator LSM)

^a^ Leerlingen uit het basisonderwijs worden niet meegenomen als onderdeel van LSM.

De gegevensverzamelingen worden geschaard onder twee onderdelen:

1. Kern van de Leefstijlmonitor (LSM-K)
  Onder de LSM‑K vallen drie gegevensverzamelingen met een vaste basis aan vragen. Dit zijn:
  de Gezondheidsenquête (GE);
  het Peilstationsonderzoek Scholieren (PEIL);
  de Health Behaviour in School-aged Children study (HBSC-onderzoek).

De GE wordt jaarlijks door het CBS uitgevoerd onder personen van 0 jaar en ouder (*N* ~ 9.500). Er wordt een random steekproef van mensen uit particuliere huishoudens getrokken uit de Basisregistratie Personen (BRP). Personen worden in eerste instantie uitgenodigd de vragenlijst via het internet in te vullen. Als ze niet via internet reageren, worden ze opnieuw benaderd via huis-aan-huisbezoeken met het verzoek de vragen uit de vragenlijst alsnog tijdens een persoonlijk interview te beantwoorden. Sinds 2018 wordt slechts een deel van de non-respondenten op de internetvragenlijst opnieuw benaderd voor een persoonlijk interview. Deze interviews worden vooral ingezet bij bepaalde groepen mensen (geïdentificeerd aan de hand van persoonskenmerken, zoals leeftijd, inkomen en migratieachtergrond) die slecht responderen via internet. Deze zogenaamde doelgroepgerichte benadering verbetert de representativiteit [6].

Voor volwassenen worden gegevens van alle bovengenoemde leefstijlthema’s uit de GE gebruikt. Voor kinderen en jeugd worden in het kader van de LSM gegevens voor de thema’s sport en bewegen, voeding, gezond gewicht en ongevallen uit de GE gebruikt. Bij kinderen onder de twaalf jaar worden de vragen door de ouders/verzorgers van het kind beantwoord. Om de vragenlijst voor iedereen begrijpelijk te maken zijn de vragen over leefstijl door Pharos gecontroleerd op taalniveau A2/B1, in samenwerking met het CBS en de andere partijen. Wanneer vragen niet aan dit niveau voldeden zijn ze indien mogelijk aangepast.

Voor roken, alcohol, drugs en seksuele gezondheid onder de jeugd (twaalf tot zestien jaar) worden gegevens uit PEIL en HBSC gebruikt. Beide onderzoeken worden om de vier jaar uitgevoerd door de Universiteit Utrecht, SCP en het Trimbos-instituut. De vraagstellingen van de thema’s die gebruikt worden voor de LSM worden tussen de beide gegevensverzamelingen afgestemd, waarmee ze voor tweejaarlijkse cijfers zorgen. In PEIL en HBSC wordt een steekproef van scholen in het regulier voortgezet onderwijs getrokken om per deelnemende school via een selectie van klassen leerlingen uit alle leerjaren van het vmbo, havo en vwo te includeren. Voor de cijfers in de LSM wordt alleen gerapporteerd over jongeren van twaalf tot en met zestien jaar (*N* ~ 7.000). Kinderen uit het basisonderwijs worden niet meegenomen voor de LSM. Er is bij de opzet van de LSM gekozen voor het gebruik van gegevens over deze thema’s op basis van PEIL en HBSC in plaats van de GE voor jeugd, omdat deze thema’s in het bijzonder van belang zijn voor de jeugd en PEIL en HBSC een grotere groep jongeren bevragen dan de GE. Doordat meer dan 90% van de jongeren van de geselecteerde klassen deelnemen aan het onderzoek worden moeilijk bereikbare groepen beter bereikt.

2. Aanvullende modules van de Leefstijlmonitor (LSM‑A)
  Onder de LSM‑A vallen gegevensverzamelingen die aanvullende en verdiepende informatie binnen een bepaald thema bieden. Deze gegevensverzamelingen zijn:
  LSM‑A Bewegen en Ongevallen;
  LSM‑A Middelen;
  LSM‑A Seksuele Gezondheid in Nederland;
  Seks onder je 25e ;
  PEIL;
  VCP.

#### LSM-A Bewegen en Ongevallen, LSM-A Middelen

De LSM‑A Bewegen en Ongevallen, en de LSM‑A Middelen worden om de twee jaar ook door het CBS uitgevoerd (*N* ~ 10.000). Voor deze gegevensverzamelingen wordt een vaste set vragen gesteld om trends te kunnen volgen die een aanvulling vormen op de LSM‑K. Daarnaast is er ruimte voor een veranderende set vragen waarmee ingespeeld kan worden op een veranderende informatiebehoefte vanuit gezondheidsbeleid en actualiteiten. Zo wordt in de LMS‑A Bewegen en Ongevallen naast beweeg- en sportgedrag ook zitgedrag uitgevraagd en worden meer vragen gesteld over de context waarin wordt bewogen en ongevallen zijn voorgekomen. In de LSM‑A Middelen wordt behalve naar roken, alcohol en drugs ook naar sportprestatieverhogende middelen (ofwel doping) gevraagd. De verdiepende vragen gaan bijvoorbeeld over problematisch alcoholgebruik en kennis van gezondheidsadviezen (zie tab. 2).

De steekproeftrekking en uitvraagmethode zijn vergelijkbaar met die van de GE. Bij het opnieuw benaderen wordt naast de huis-aan-huisbezoeken echter ook een deel telefonisch benaderd. Sinds 2019 wordt ook hier de doelgroepgerichte benadering ingezet. De leeftijdsgroep waaronder de vragenlijsten worden uitgezet betreft bij de LSM‑A Bewegen en Ongevallen personen van 0 jaar en ouder. Bij de LSM‑A Middelen is dit 15 jaar en ouder. Bij de LSM‑A Bewegen en Ongevallen is de steekproef voor 0‑ tot 17-jarigen opgehoogd en bij de LSM‑A Middelen is de steekproef voor 20- tot 34-jarigen opgehoogd om de power van de voor de betreffende thematiek relevante leeftijdsgroepen te vergroten.

#### LSM-A Seks onder je 25e en LSM-A Seksuele Gezondheid in Nederland

De LSM‑A Seksuele gezondheid in Nederland (*N* ~ 17.000 18- tot 79-jarigen) en de LSM‑A Seks onder je 25e (*N* ~ 20.000 18- tot 24-jarigen) zijn vanaf 2017 met elkaar geïntegreerd, omdat de doelgroepen van beide onderzoeken deels overlappen. Dat wil zeggen dat de vragenlijsten van de twee onderzoeken vergelijkbaar zijn gemaakt en de gegevens in dezelfde periode zijn verzameld. De vragenlijst dekt een breed scala van aan seksualiteit gerelateerde onderwerpen, zoals seksueel gedrag, risico op soa’s en onbedoelde zwangerschap en seksueel geweld. Bij beide onderzoeken is gebruikgemaakt van een random steekproef uit de BRP. Bij de LSM‑A Seks onder je 25e zijn ook jongeren via scholen benaderd. De BRP-steekproefpersonen zijn uitgenodigd om via internet deel te nemen. Personen die niet via internet reageren en boven de 25 jaar zijn kregen een papieren vragenlijst thuis gestuurd. In 2022 worden deze gegevensverzamelingen herhaald.

#### PEIL

Bij PEIL (zie ook LSM-K) worden naast basisvragen om prevalentiecijfers van roken, alcohol- en druggebruik en veilige seks te verkrijgen, ook verdiepende vragen gesteld. Deze vragen geven informatie over bijvoorbeeld de manier waarop de jeugd aan rook- en alcoholproducten komt of over hun attitude over middelengebruik.

#### VCP

De VCP geeft inzicht in de consumptie van voedingsmiddelen, de inname van macro- en microvoedingsstoffen en de inname van potentieel schadelijke chemische stoffen, en in de ontwikkelingen hiervan (trends), en informatie over de milieudruk. De VCP 2012–2016 heeft gebruikgemaakt van een consumentenpanel van 1‑ tot 79-jarigen (*N* ~ 4.000) woonachtig in Nederland. Bij hen is de consumptie van voedingsmiddelen nagevraagd met behulp van een computergeassisteerde 24-uursvoedingsnavraagmethode, waarin heel specifiek is nagevraagd welke producten er zijn geconsumeerd en hoeveel.

De VCP en de GE vullen elkaar aan op het gebied van voeding. De VCP brengt gedetailleerd in kaart (op voedingsmiddelen- en nutriëntenniveau) wat Nederland eet en drinkt. De GE geeft enkel inzicht in een aantal voedingsmiddelen (groente, fruit en vis) aan de hand van algemene vragen. De VCP brengt in zijn algemeenheid de voeding beter in beeld, maar wordt bij minder mensen én minder vaak uitgevoerd dan de GE. Daarom is het op basis van de GE makkelijker uitspraken te doen over trends en verschillen tussen specifieke groepen. Door de verschillen in de methodiek zijn de cijfers over consumptie van groente en fruit echter hoger op basis van de GE dan op grond van de VCP. Bij de duiding van deze cijfers wordt hier rekening mee gehouden.

### Zijn de cijfers representatief voor de Nederlandse bevolking?

Om cijfers te verkrijgen die representatief zijn voor de Nederlandse bevolking worden de prevalentiecijfers bij alle gegevensverzamelingen van de LSM gecorrigeerd met een weegfactor [5]. De weegfactor corrigeert voor de verschillen tussen de samenstelling van de respons uit de steekproef en de samenstelling van de Nederlandse bevolking op basis van verschillende kenmerken, zoals geslacht, leeftijd en inkomen. Dit zorgt ervoor dat de cijfers een zo goed mogelijk beeld geven van de Nederlandse bevolking.

## Hoe verhoudt de LSM zich tot lokale en internationale gegevensverzamelingen?

Een aantal gegevensverzamelingen binnen de LSM is geharmoniseerd met lokale en/of internationale gegevensverzamelingen. De vragen over roken en alcohol (met uitzondering van 2016), lengte, gewicht en bewegen in de GE zijn geharmoniseerd met de vragen over deze onderwerpen in de Gezondheidsmonitor (GM) Volwassenen en Ouderen van de GGD’en. Deze verzamelt eens in de vier jaar gegevens op regionaal en lokaal niveau ter ondersteuning van lokaal, regionaal en landelijk gezondheidsbeleid [7]. Door deze harmonisatie zijn de landelijke cijfers vergelijkbaar met regionale en lokale cijfers voor deze onderwerpen. Naast de GM Volwassenen en Ouderen wordt ook elke vier jaar een GM Jeugd uitgevoerd, die eveneens cijfers levert op lokaal en regionaal niveau. De doelgroep betreft leerlingen van het tweede en vierde leerjaar van het reguliere voortgezet onderwijs. De GM Jeugd bevraagt deels dezelfde thema’s als PEIL en HBSC, en daarom worden de vragenlijsten zo goed mogelijk op elkaar afgestemd.

De GE wordt ook gebruikt in een internationale context. De European Health Interview Survey (EHIS) wordt eenmaal in de zes jaar uitgevoerd in opdracht van de Europese Commissie. De GE levert cijfers aan op basis van de bestaande vragen of met een aantal aanpassingen om aan de eisen van de EHIS te voldoen [8].

Het HBSC-onderzoek is ook een bron die in een internationale context wordt gebruikt [9]. Het HBSC-consortium bestaat uit vijftig landen uit Europa en Noord-Amerika, en rapporteert aan het Regionale kantoor van de Wereld Gezondheidsorganisatie (WHO Regional Office for Europe).

## Wat levert de LSM op voor gezondheidsbeleid, -praktijk en -onderzoek?

De cijfers die uit de kern- en aanvullende modules van de LSM komen dienen in de eerste plaats ter ondersteuning van het landelijk gezondheidsbeleid en worden in verschillende producten voor het ministerie van VWS gepubliceerd. Dit betreft rapportages, zoals de Nationale Drug Monitor [10], Letsels 2020: Kerncijfers LIS [11] en Seksuele Gezondheid in Nederland [12]. Ook zijn er websites opgezet specifiek voor het ontsluiten van de cijfers van de LSM. Dit zijn www.leefstijlmonitor.nl, www.wateetnederland.nl, www.sportenbewegenincijfers.nl en www.nationaledrugsmonitor.nl. Daarnaast zijn de cijfers terug te vinden op de websites van de verschillende consortiumpartners en op websites over de staat van de volksgezondheid en zorg in Nederland van het ministerie van VWS, www.volksgezondheidenzorg.info en www.staatvenz.nl. Deze webpagina’s worden overigens ook geregeld gebruikt door personen die in de praktijk op het gebied van leefstijl werken, zoals fysiotherapiepraktijken die willen weten welke doelgroepen achterblijven in beweeggedrag, zodat ze hun interventies daar op kunnen richten.

Derden kunnen de gegevensverzamelingen of maatwerkanalyses van de LSM voor onderzoeksdoeleinden aanvragen. Op de webpagina ‘Data aanvragen’ van www.leefstijlmonitor.nl is te vinden bij welke partij en onder welke voorwaarden de gegevensverzamelingen op te vragen zijn. Een aantal gegevensverzamelingen (GE en de LSM-A’s Middelen, Bewegen en Ongevallen en Seksuele Gezondheid in Nederland) is beschikbaar via het CBS (dat wil zeggen tegen betaling via Remote Access). Daardoor zijn de gegevensverzamelingen te koppelen aan andere databronnen bij het CBS, zoals registraties met betrekking tot medicatie en zorg, uitkeringen en woon- of buurtkenmerken. Dit biedt extra mogelijkheden voor verdiepend onderzoek.

## Voorbeelden van cijfers voor gezondheidsbeleid

Hieronder volgt een aantal voorbeelden van cijfers en trends van 2014–2020 die specifiek worden gebruikt ter ondersteuning van het gezondheidsbeleid, zoals het monitoren van de doelstellingen in het Nationaal Preventieakkoord en het Sportakkoord [13, 14]. De cijfers van 2020 moeten met enige terughoudendheid worden geïnterpreteerd vanwege de COVID-19-pandemie. De pandemie kan invloed hebben gehad op het gedrag en de gezondheid van de respondent zelf. Daarnaast zijn de persoonlijke interviews voor een deel van het jaar weggevallen vanwege de geldende overheidsmaatregelen. Hiervoor is gecorrigeerd via een weegmodel, zodat de cijfers vergelijkbaar zijn met voorgaande jaren [15]. Voor het thema sport en bewegen corrigeert dit model de cijfers echter onvoldoende, waardoor de cijfers positiever uitvallen dan de werkelijkheid [16].

### Nationaal Preventieakkoord en ander gezondheidsbeleid

In het Nationaal Preventieakkoord zijn verschillende ambities uitgesproken over de thema’s roken, overgewicht en alcohol, zoals ‘In 2040 rookt minder dan 5% van de volwassenen (> 18 jaar)’. Figuur 1 bevat trends over de periode van 2014–2020 waarmee dergelijke ambities kunnen worden gemonitord. Uit de figuur blijkt dat het aandeel volwassenen dat rookt tussen 2014 en 2020 is afgenomen van 26% naar 20%. Daarnaast is het aandeel volwassenen dat overmatig drinkt afgenomen van respectievelijk 10% naar 7%. Zwaar drinken is gelijk gebleven (±8%). Het aandeel volwassenen met matig overgewicht en obesitas is in deze periode redelijk stabiel gebleven, met respectievelijk ongeveer 35% en 14%.

**Figure 1:**
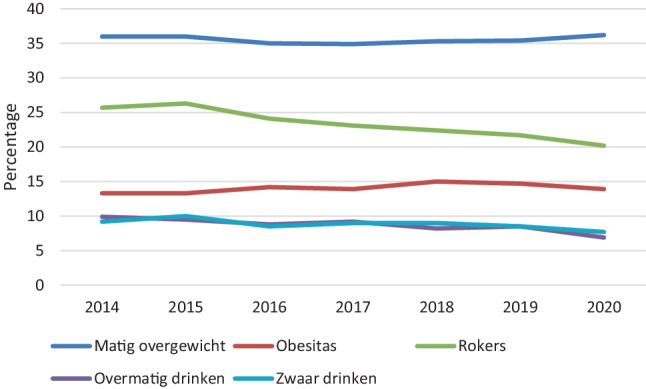

Naast deze thema’s is de voedselconsumptie een belangrijk onderdeel van het preventiebeleid ter voorkoming van chronische ziekten. Op basis van VCP/Leefstijlmonitor, RIVM 2012–2016, blijkt dat onder 1‑ tot 79-jarigen gemiddeld 131 gram groente en 113 gram fruit per dag wordt gegeten. Circa 1 op de 6 van de volwassenen eet de door de Gezondheidsraad aanbevolen 200 gram of meer groente of fruit.

### Sportakkoord en blessurepreventie

**Figure 2:**
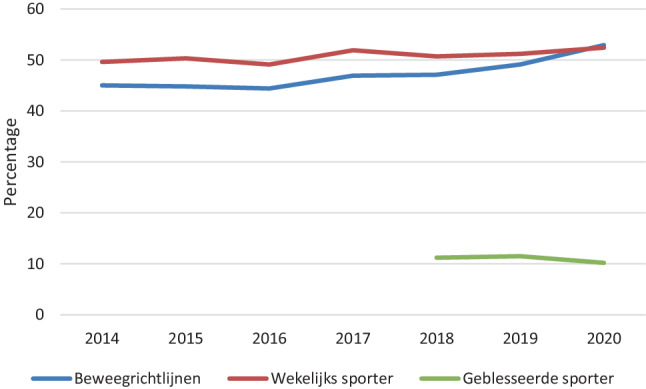

Het aandeel personen dat voldoet aan de beweegrichtlijnen en dat wekelijks sport wordt onder andere gemonitord om de voortgang van de uitvoering van het Sportakkoord te bepalen. In fig. 2 is te zien dat het aandeel volwassenen dat wekelijks sport tussen 2014 en 2020 net boven de 50% schommelt. Het aandeel dat aan de beweegrichtlijnen voldoet, is in die periode gestegen van 45% naar 53%. Het aandeel geblesseerde sporters in de afgelopen 3 maanden wordt gebruikt als input voor het blessurepreventiebeleid en is tussen 2018 en 2020 redelijk stabiel gebleven (~ 10%).

### Drugsbeleid en beleid rond seksuele gezondheid en letsel- en valpreventie

Figuur 3 bevat een aantal voorbeelden van LSM-cijfers ter ondersteuning van de vorming van het drugsbeleid, beleid rondom seksuele gezondheid en letsel-en valpreventie.

**Figure 3:**
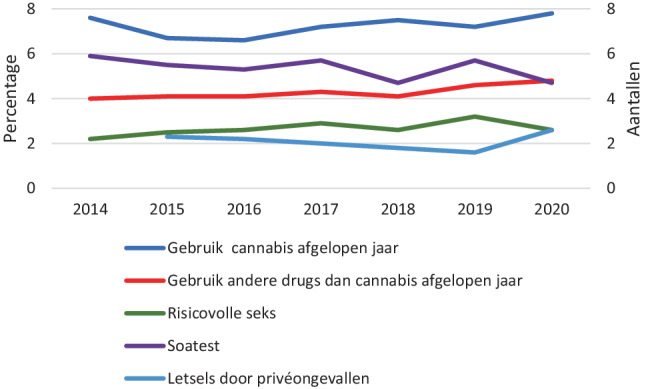

De cijfers laten zien dat het gebruik van cannabis en andere drugs tussen 2014 en 2020 in het voorgaande jaar bij volwassenen redelijk stabiel is gebleven (respectievelijk ongeveer 7% en ongeveer 4%).

Het aandeel volwassenen dat risicovolle seks (dat wil zeggen laatste sekscontact met losse/betaalde sekspartner én onbeschermd (zonder condoom)) heeft gehad en zich heeft laten testen op seksueel overdraagbare aandoeningen is in diezelfde periode nagenoeg stabiel gebleven. De percentages liggen respectievelijk rond de 2 tot 3% en de 5 tot 6%.

Het aantal letsels per persoon als gevolg van een privéongeval in het voorgaande jaar is tussen 2015 en 2020 ook nagenoeg stabiel gebleven en ligt rond de 2 tot 3 letsels.

### Gezondheidsverschillen

**Figure 4:**
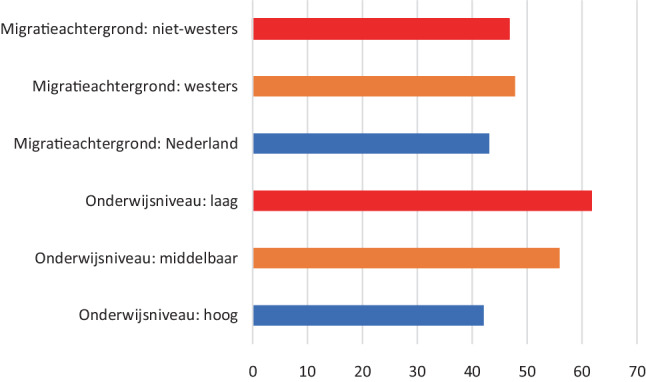

Het terugdringen van gezondheidsverschillen staat hoog op de agenda bij het ministerie van VWS [17]. Binnen de LSM kunnen alle indicatoren uitgesplitst worden naar verschillende achtergrondkenmerken (zie tab. 2 voor een overzicht), zoals onderwijsniveau en migratieachtergrond, waardoor gekeken kan worden naar gezondheidsverschillen. Als voorbeeld zijn in fig. 4 voor het jaar 2020 de verschillen weergegeven in overgewicht tussen personen met verschillende migratieachtergronden en onderwijsniveaus. Personen met een niet-westerse migratieachtergrond hebben vaker overgewicht dan personen zonder een migratieachtergrond (48% versus 44%). Personen met een laag en middelbaar onderwijsniveau hebben vaker overgewicht dan personen met een hoog onderwijsniveau (respectievelijk 61%, 56% en 43%) (fig. 4). Dergelijke gezondheidsverschillen zijn te zien bij vrijwel alle leefstijlfactoren en daarom is het belangrijk om ze in kaart brengen.

## Wat zijn de sterke en zwakke punten van de LSM?

Een van de sterke punten van de LSM is dat verschillende instituten werkzaam op het gebied van leefstijl hun krachten en expertise hebben gebundeld om de samenhang tussen de gegevensverzamelingen te borgen. Zo worden in één vragenlijst (GE) alle leefstijlfactoren uitgevraagd, waardoor naar de onderliggende verbanden gekeken kan worden in samenhang met gezondheid. Daarnaast is een aantal gegevensverzamelingen beschikbaar bij het CBS, waardoor diepgaander onderzoek mogelijk is door gegevensverzamelingen van de LSM te koppelen aan andere databronnen. Deze onderzoeken bieden een schat aan informatie voor het vormen van gezondheidsbeleid. Zo is het monitoren van informatie waarbij leefstijl naar onderwijsniveau en inkomen wordt uitgesplitst bijvoorbeeld van belang bij het stellen van doelen binnen het Nationaal Preventieakkoord. Een ander pluspunt is dat binnen alle onderzoeken van de LSM wordt ingezet op het verhogen van de representativiteit van de steekproef. Dit wordt bijvoorbeeld gedaan door de uitnodigingen te richten op bepaalde doelgroepen die minder goed responderen (een doelgroepgerichte benadering).

Naast sterke punten kent de LSM ook zwakkere punten. De gegevensverzamelingen van de LSM zijn cross-sectioneel. Dit is geschikt voor het doel waarvoor ze is opgezet, maar minder bruikbaar voor vragen over bijvoorbeeld veranderingen in leefstijl voorafgaand aan gezondheidsklachten. Daarnaast zijn de gegevens voornamelijk verzameld met vragenlijsten. Hoewel er wordt geïnvesteerd in het waarborgen van de representativiteit [6], blijft het een uitdaging om de verschillende gegevensverzamelingen volledig representatief te laten zijn doordat de respons over tijd afneemt en bepaalde doelgroepen slecht responderen, zoals personen met een migratieachtergrond en een laag inkomen [18]. Er zijn verschillende acties mogelijk om te compenseren voor de afnemende respons in bepaalde doelgroepen en om de representativiteit te optimaliseren, bijvoorbeeld het inzetten van incentives (dat wil zeggen een cadeaubon van 5 euro of het verloten van een IPad) en zoals eerder gezegd de inzet van de doelgroepgerichte benadering [6]. Daarnaast worden de cijfers gewogen naar kenmerken uit de bevolking [5]. Bepaalde doelgroepen zitten echter niet in de steekproef, zoals ouderen in een verpleeghuis en personen met een verstandelijke beperking. Bovendien kan bij de steekproeftrekking en herbenadering niet op alle kenmerken van een persoon geselecteerd worden (zoals lage opleiding, laaggeletterdheid en gezondheid).

Ondanks de beperkingen hebben we dankzij de LSM een goede indicatie van de algemene gezondheidstrends en kunnen we verschillen tussen bepaalde doelgroepen toetsen. Bij gebruik van de cijfers moet hier echter rekening mee worden gehouden. Om de representativiteit verder te verbeteren onderzoekt het consortium de mogelijkheden om andere methoden toe te voegen om specifieke doelgroepen beter te kunnen includeren. Bij het afnemen van de vragenlijst door middel van persoonlijke interviews kunnen we denken aan het inzetten van interviewers die de culturele achtergrond van de respondent delen. Een andere mogelijkheid is het inzetten van meer kwalitatieve vormen van onderzoek bij specifieke doelgroepen, zoals personen die in probleemwijken wonen, scholieren van het middelbaar beroepsonderwijs (mbo) of mensen met een verstandelijke beperking. Het consortium bekijkt bij dergelijke opties in hoeverre ze in te passen zijn in bestaande monitoringsstructuren, of dat aanvullende onderzoeken nodig zijn.

Het consortium werkt ook aan het verbeteren van methoden om leefstijl te monitoren. Zo wordt op nationaal en Europees niveau onderzocht of bewegen met een objectieve maat gemonitord kan worden [19] en wordt gekeken of de gegevens over voeding verzameld kunnen worden met een app waarbij deelnemers hun gegeten voedingsmiddelen kunnen scannen [20]. Ten slotte levert vragenlijstonderzoek vooral nuttige informatie op over gedragingen die van toepassing zijn op grote groepen mensen. Voor laagprevalent gedrag, zoals drugsgebruik en het hebben van sportblessures, is het informatief om te monitoren dat dit relatief weinig voorkomt. Om handvatten voor preventie te bieden is het echter nodig om aanvullend onderzoek te doen onder de groep mensen met laagprevalent gedrag.

## Conclusie

De LSM omvat een rijke verzameling aan gegevens van verschillende leefstijlfactoren. De LSM zorgt voor efficiëntie en samenhang tussen verschillende gegevensverzamelingen dankzij de samenwerking tussen partijen op het gebied van leefstijl en gezondheid. De gegevens worden veelvuldig gebruikt ter ondersteuning van het gezondheidsbeleid, zoals het monitoren van de doelen binnen het Nationaal Preventieakkoord en het Sportakkoord. De gegevensverzamelingen zijn op te vragen voor onderzoeksdoeleinden en kunnen vaak gekoppeld worden aan andere databronnen, waarmee een grote verscheidenheid aan gezondheidsvraagstukken kan worden onderzocht.
